# Gesture-Based Interactions: Integrating Accelerometer and Gyroscope Sensors in the Use of Mobile Apps

**DOI:** 10.3390/s24031004

**Published:** 2024-02-04

**Authors:** Sergio Caro-Alvaro, Eva Garcia-Lopez, Alexander Brun-Guajardo, Antonio Garcia-Cabot, Aekaterini Mavri

**Affiliations:** 1 Universidad de Alcalá, Departamento de Ciencias de la Computación, 28805 Madrid, Spain; sergio.caro@uah.es (S.C.-A.); alexander.brun@edu.uah.es (A.B.-G.); a.garciac@uah.es (A.G.-C.); 2 Cyprus Interaction Lab, Department of Multimedia and Graphic Arts, Cyprus University of Technology, 30 Archbishop Kyprianou Str., Limassol 3036, Cyprus; aekaterini.mavri@cut.ac.cy

**Keywords:** user experience, HCI, mobile apps, gyroscope, accelerometer, gesture-based interactions

## Abstract

This study investigates the feasibility and functionality of accelerometer and gyroscope sensors for gesture-based interactions in mobile app user experience. The core of this innovative approach lies in introducing a dynamic and intuitive user interaction model with the device sensors. The Android app developed for this purpose has been created for its use in controlled experiments. Methodologically, it was created as a stand-alone tool to both capture quantitative (time, automatically captured) and qualitative (behavior, collected with post-task questionnaires) variables. The app’s setting features a set of modules with two levels each (randomized presentation applied, minimizing potential learning effects), allowing users to interact with both sensor-based and traditional touch-based scenarios. Preliminary results with 22 participants reveal that tasks involving sensor-based interactions tend to take longer to complete when compared to the traditional ones. Remarkably, many participants rated sensor-based interactions as a better option than touch-based interactions, as seen in the post-task questionnaires. This apparent discrepancy between objective completion times and subjective user perceptions requires a future in-depth exploration of factors influencing user experiences, including potential learning curves, cognitive load, and task complexity. This study contributes to the evolving landscape of mobile app user experience, emphasizing the benefits of considering the integration of device sensors (and gesture-based interactions) in common mobile usage.

## 1. Introduction

Modern smartphones have a diverse array of internal sensors, spanning from capacitive sensors (responsible for tactile screen functionality), to GPS, fingerprint readers, iris scanners, and proximity or brightness sensors. Gyroscope and/or accelerometer sensors are commonly included among device capabilities.

**Figure 1:**
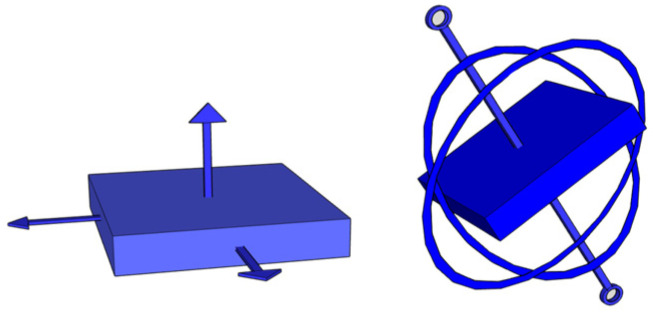
Conceptual design of the accelerometer (**left**) and gyroscope (**right**) sensors.

The accelerometer serves as a sensor capable of detecting the direction and intensity of the device’s movement. Technically, it measures the linear acceleration of movement (i.e., gravity) [1]. However, it is not a very accurate sensor for determining orientation, as it is usually built as a basic hardware component with electrically sensitive elements and capacitors [2]. The gyroscope comes into play for greater accuracy, allowing the rotation of the device around its axes to be calculated in a simpler way (by calculating the angular rate of rotation) [1]. In summary (see Figure 1), the accelerometer sensor measures the speed and direction of movement of the device and the gyroscope sensor measures the rotation of the device about the Cartesian axes.

The vast majority of available devices on the market today have both the gyroscope and the accelerometer sensor pair, although it is true that low-end devices may not have the gyroscope, since it requires hardware with a higher unit price than the accelerometer sensor hardware. We can also find some devices, such as the Motorola Moto G3 (launched in 2015), that included two accelerometers (without a gyroscope), with the aim of improving the precision that a single accelerometer would provide [3].

While it is true that Google does not require brands to include accelerometers when certifying a mobile device as compatible with the Android operating system, it is a hardware feature that Google strongly recommends [4]. The gyroscope sensor is also an optional component for certification.

On the other hand, Apple has included an accelerometer in its devices since the first iPhone model (launched in 2007) [5]. In addition to the accelerometer, Apple has included a gyroscope sensor in all new iPhone models since the iPhone 4 was launched, in 2010 [6].

The main visible usage of these sensors, when integrated in a smartphone, is that the device automatically adjusts the orientation of the User Interface (UI) to the physical position of the device. This means that, when the device is rotated from vertical to horizontal, or vice versa, the UI is set in the corresponding landscape or portrait mode, according to the values obtained from the accelerometer and gyroscope sensors, if present. The aim of this automatic adaptation is to provide the user with a more comfortable experience with the device.

Our research is therefore linked to the Human–Computer Interaction (HCI) field, devoted on enhancing products by understanding how individuals interact with computers [7]. It is commonly applied to enhance the user experience (UX) with specific software products. UX explores how individuals perceive and respond to software usage to enhance product design [8,9,10]. Usability, a component of UX, is commonly used to assess the user experience [8]. ISO defines usability as “the effectiveness, efficiency, and satisfaction with which specified users achieve specified goals in particular environments” [11].

In this study, we run a pilot experimentation to assess the feasibility and functionality of gesture-based interactions in mobile apps, in which traditional touch-based interactions prevails. When these sensors are integrated into a smartphone, there are a number of utilities that are available both to the operating system and to the application developers:

- Automatic UI orientation. Previously introduced, it allows the UI to adapt its displayed content according to the detected orientation of the device.
- Pedometer. As these sensors can measure movement, they are used as pedometers to count steps and allow users to make a detailed analysis of calories burned, distance, etc. As a result, these sensors are now widely used in health and sports applications.
- Image stabilization. Prevents the quality of the captured image from being affected by the shaking of the user’s hand. The effect of vibrations is reduced both in photos and videos.
- Inertial Navigation. Thanks to the information obtained from these sensors, GPS can still provide navigation information in the event of a loss of GPS service or cellular coverage (e.g., inside tunnels or buildings).
- Games (motion control). It allows game controls to be triggered by gestures—for example, using the phone as a steering wheel while driving a car. The game replicates the movements/gestures made with the phone into the game, without tapping on the screen (which reduces the visible percentage of the screen, as the hands block part of the view).
- Gesture-based interactions. This involves interacting with the UI through motion/gesture detection, and is perhaps the least implemented application in mobile apps [12,13]. Informally, this could be seen as the non-game version of interacting with the device (i.e., using the movement of the device as a game control). Compass or level applications are the most typical examples of usage.

**Figure 2:**
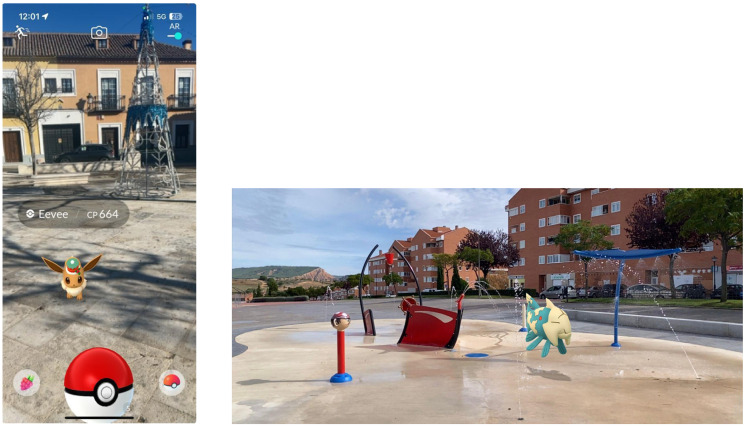
Details of Pokémon GO Augmented Reality interface and the real world.

Of the above applications, using accelerometers and gyroscopes as in-game controllers has been the most common implementation in recent years. The popular mobile game “Pokémon GO” (released in 2016) uses this technology to immerse the player, using the device’s camera as a window into a parallel world. Using accelerometer and gyroscope sensors, it allows the players to “see” the world around them by moving the device, without needing touch interaction (see Figure 2). This combination (real world combined with virtual objects, interacted in real time) is defined as Augmented Reality (AR) [14]. This popularity is reflected in the fact that in the following year (2017), Apple launched ARKIT and Google released ARCore, both development tools that are specifically designed to facilitate the development of AR applications. It should be noted that to achieve the immersion provided by AR is where accelerometer and gyroscope sensors come into play as essential sensors [15].

**Figure 3:**
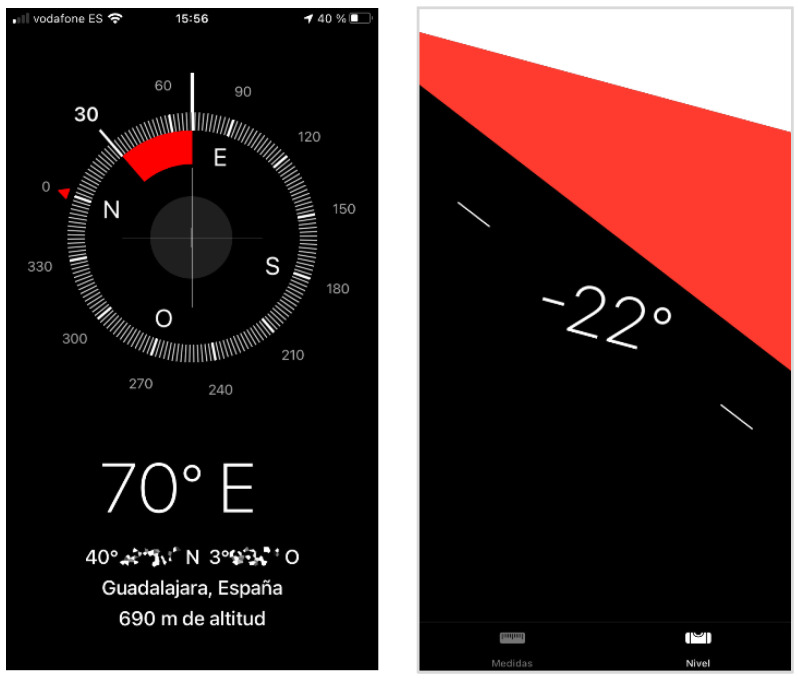
Detail of the compass (**left**) and level (**right**) apps on the iPhone (own sources).

Beyond the gaming industry, many applications that use the accelerometer and gyroscope sensors can be found on current smartphones. The most characteristic applications of this type of sensors are compass apps (providing orientation) (see Figure 3, left) or level apps (measuring surface’s horizontality or verticality) (see Figure 3, right).

Natively, iPhone devices also include two features related to these sensors: “shake to undo” and “shake to shuffle”. The first one, “shake to undo”, allows the user to delete the last thing he/she typed by giving the iPhone a quick shake (user is always asked for confirmation). It has been available since iOS 3.0 (build 7A341, 2009), although without official confirmation of its existence [16,17]; and at least until iOS 9 (build 13A340, 2015) [18]. The second one, “shake to shuffle”, allows the user to skip the current playing song by shaking the device (also since the build 7A341, 2009) [19].

Alternatively, there are several applications in both the Android and iOS stores that use the accelerometer and/or gyroscope sensors for their basic operation, such as “MouseMote AirRemote” (which allows using the smartphone as a remote mouse for the computer) [20] or “SkyView” (star map that updates visible sky based on device rotation and orientation) [21].

This clearly demonstrates the opportunities of integrating accelerometers and gyroscopes in the development of mobile applications. However, interacting with UIs through motion and/or gesture detection, i.e., gesture-based interactions [22], is a research area with very little coverage [23]. According to Sun et al. [12] this could be due to unknown hardware availability, difficulties to transform abstract gestures into meaningful ones or difficulties delivering gestures to generic scenarios. Gesture-based interactions could be seen as non-regular tasks with mobile apps. Therefore, a higher cognitive load is expected for users when engaging with novel tasks (as derived from Banu, Al Siyabi and Al Minje [24], and Kosch et al. [25]).

From our proposed research line, however, the focus of the literature diverges. Here, we are centered on the interaction with the device’s UI through the accelerometer and the gyroscope sensors. Nonetheless, the main research found is focused on more specific areas, such as the classification of motion and movement [13,26,27], the analysis of road conditions [28,29], medical applications [30,31], and even cyber security [32]. Because of the relatively innovative nature of our research, the focus of this study will be on the detailed analysis of how traditional (touch-based) interaction can be converted to gesture-based interactions, so that the User Experience with UIs will be improved.

According to Sun et al. [12], there are three main types of gestures to interact with electronic devices (either smartphones, smartwatches, glasses or mixed devices): Device based, touch based and vision based. Device-based gestures imply physically moving the device (driven by three key features: “Ensure basic functionality”, “enhance accessibility” and “assign meaning and value”); the touch-based ones are the traditional way of interact, using the hand to interact with the screen; and the vision-based ones imply mid-air gestures made with body parts, mainly with the user’s hand or head.

It is a fact that current smartphones have larger and larger screens. This makes it difficult for the user to interact with the device with only one hand. The work of Chang et al. [33] proposed that an application would change the available UI so that it can be reached with a finger without the need to tilt the device or force the position of the hand. This was performed by using the accelerometer and the gyroscope for detecting when the mobile enters into a forced position. They designed three novel interactions: “TiltSlide”, “TiltReduction” and “TiltCursor”. In “TiltSlide”, the screen is moved by the user to a more finger-accessible zone (here, the UI keeps moving with sensors’ inputs until one of the UI corners moves to the center of the screen). In “TiltReduction”, the UI is zoomed-out (i.e., size reduction) and adjusted to a more accessible zone. Finally, in “TiltCursor”, the user does not directly interact with the UI; instead, the user’s finger moves a virtual pointer through the UI. Looking at their results, only “TiltReduction” interaction has better results than touch-based interactions.

A more recent study, by Lee et al. [34], proposed alternative hardware to replace the most basic interactions (that is, selection and movement) by detecting hand pressure on this alternative hardware sensors.

As aforementioned, AR is currently experiencing a growth in popularity. This is also reflected in the scientific literature. There are several studies, such as Huang, Li and Hui [35], Vatavu [36], Di Geronimo et al. [37] or Mich et al. [38]; that use accelerometer and gyroscope sensors and hand gestures (detected by the camera) to find new ways of interacting with non-gaming AR applications. The goal is to make AR apps more intuitive for the users.

However, even though it is limited, it is possible to find some literature that is more in line with what is proposed in our research.

In 2009, the work of Boring, Jurmu and Butz [39] used one of the first generation of smartphones (previous to the iPhone/Android) as a pointer on a plasma screen. Interaction was based on detecting information from the accelerometer and gyroscope sensors and translating them into the remote mouse motion.

In 2016, Serackis et al. [40] presented a study that investigated replacing traditional smartphone keyboards with another keyboard, also virtual, but interacted through tilting and rotating the phone. This proposed keyboard translates the movements obtained through the accelerometer and gyroscope sensors into a pointer across a virtual keyboard. However, this was only a prototype. The authors did not include how this compares to using a traditional keyboard, as it was presented as a very specific topic without proper research to compare with. Nevertheless, they stated that all participants were able to successfully complete all the required tasks.

In 2017, Yu et al. [41] proposed a new way of interacting with smartphones. They took advantage of the boom in popularity of smartwatches. They used the accelerometer sensor of the smartwatch itself to detect when the device is moving in a certain direction. They captured these movements and translated them to interactions on the smartwatch app. The authors even demonstrated multi-device compatibility with an example of using these detected movements on the smartwatch in a simple navigation through a list of items displayed on the smartphone.

Given the lack of studies on the use of accelerometer and gyroscope sensors in interacting with UIs, this research serves as a novel approach to explore the impact of gesture-based interactions on smartphone apps. Overall, this research contributes to a better understanding of the complex dynamics between gesture-based interactions and user experiences, with implications for the design and evaluation of future mobile applications using sensors.

The rest of this paper is organized as follows: Section 2 presents the proposed Android app, how it works and how the sensors are integrated in the app. In Section 3, the preliminary results are illustrated by showing the outcomes from a pilot experiment. Finally, Section 4 presents the discussion, and Section 5 shows conclusions and future works.

## 2. Materials and Methods

To investigate the potential enhancement of mobile app user experience through accelerometer and gyroscope sensors, we developed an Android app designed for its usage in controlled experiment sessions with participants. The following sections detail the design and development procedures employed in the creation of this app.

### 2.1. Description of the Mobile Application

**Figure 5:**
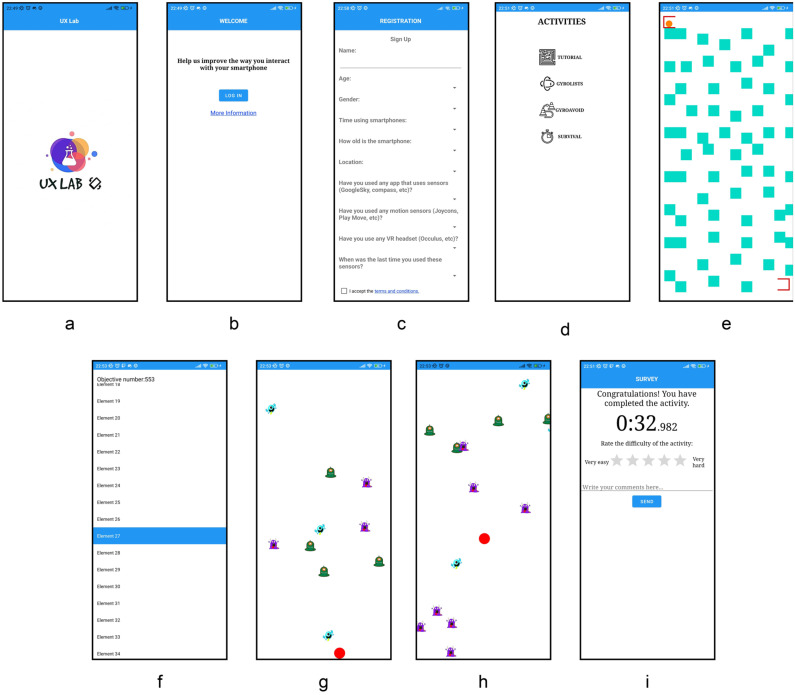
Windows workflow of the application: (**a**) Splash screen, (**b**) login page, (**c**) registration page, (**d**) activities screen, (**e**) tutorial screen, (**f**) gyrolist screen, (**g**) gyroavoid screen, (**h**) survival screen, and (**i**) survey screen.

The app developed comprises various modules with levels that use the gyroscope and accelerometer sensors to investigate gesture-based and traditional-based interactions through the UI. The utilization of these interactions in the available levels enables the autonomous collection of quantitative data, in the form of required time to complete a given task. Qualitative data, in the form of user behavior, are collected with an in-app post-task questionnaire. The application has been designed as a stand-alone app, i.e., no human is needed for data collection. The experiment’s instructor should be in charge of guiding participants on performing the selected tasks. Figure 5 (at the end of this section) shows the main windows and the basic workflow of the application.

The development of the activity encompasses various aspects and methodologies. The application features an integrated login system with Google, using Google Firebase as the platform for managing user authentication, as seen in Figure 5b. Upon initial registration, users are redirected to an initial questionnaire designed to collect demographic data and information about previous experiences with sensors. This questionnaire includes questions related to the use of sensors integrated into the phone (noticeable in Figure 5c). Users are also queried about the use of motion sensors found in video game consoles, such as “Joy-Cons”, and virtual reality headsets, such as “Oculus VR” or “PlayStation VR”. 

**Figure 4:**
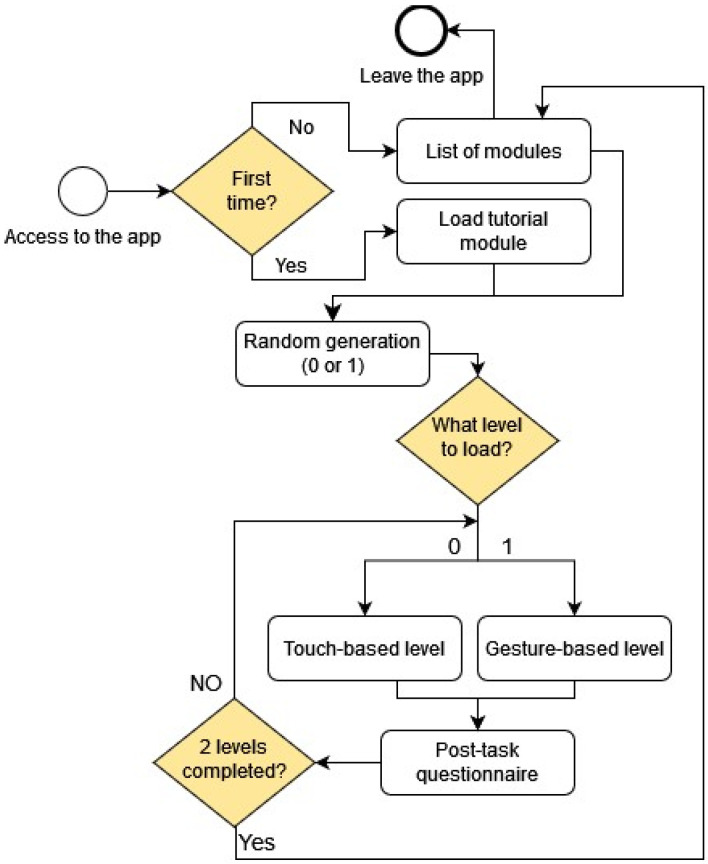
Flow of actions with the app modules.

As seen in Figure 4, the logical structure of the application’s levels is designed to ensure an unbiased exploration of user interactions. There are four available modules in the app: “Tutorial”, “GyroList”, “GryoAvoid” and “Survival”. Each module within the application is composed of two distinct levels, each representing a set of challenges related to a device sensor or touch interactions. Importantly, the first level of every module is intentionally selected at random for each user, effectively minimizing potential learning effects that could arise with device-based and traditional touch-based interactions. By introducing this element of unpredictability in level selection, we aim to capture experiences free from the influence of pre-existing patterns or expectations.

Once the initial questionnaire is completed, users are directed to the “Tutorial” module, (Figure 5e), designed to familiarize them with the use of the sensors. This activity involves guiding a ball from a starting point to a goal while avoiding collisions with walls along the way, simulating a maze. The tutorial provides an interactive introduction to the accelerometer and gyroscope sensors, thus helping users understand how to use them while controlling the ball’s movement.

Upon completion of the tutorial, new modules are unlocked (Figure 5d), with levels that can be performed either in a *traditional* way (i.e., using the touch controls) or by using the device sensors, depending on the level. One of these modules is called “GyroLists”, (Figure 5f), where the goal is to navigate through a list of hundreds of elements until reaching a predefined element. In the *traditional* level, users manually scroll and select the target element by swiping with their finger. In the sensorized level, scrolling is performed automatically through an “autoscroll”, and the element is selected automatically.

Another module, named “GyroAvoid”, (Figure 5g), involves maneuvering a ball to avoid falling blocks descending from the top of the screen. In this scenario, the user moves the ball solely left and right using his or her finger in the *traditional* form. However, in the gesture-based form, the movement is achieved by tilting the phone in the desired direction.

In addition, there is a “Survival” module (Figure 5h), which also involves controlling a ball to evade falling blocks. Unlike the previous activity, in “Survival” the blocks come from all directions, so the movement is no longer limited to just left and right; instead, the ball can move in any direction, similar to the tutorial activity. As with the other activities, there is both a *traditional* form, where the ball’s movement is achieved using the finger (i.e., touch based), and with the device sensors (i.e., gesture based), where, once again, tilting the phone in the desired direction achieves the movement.

It should be highlighted that, in the “Tutorial” and “GyroLists” modules, the primary objective for users is to maximize speed, with faster completion times indicating a better user performance. On the other hand, “GyroAvoid” and “Survival” modules present users with a different challenge: The objective here is time endurance, skipping through as many elements as possible. In this context, higher completion times denote a better user performance. These scenarios aim to capture a comprehensive spectrum of user behaviors on the dynamics of gesture-based and touch-based interactions and their impact on mobile app usage.

After completing each module level, users are prompted to take a satisfaction questionnaire (Figure 5i). This questionnaire displays the time taken to complete the activity, asks users to rate the difficulty of the activity in its respective mode, and provides an opportunity to add additional comments about their experience. These data, like those from the initial survey, are stored in different collections in Google Firebase’s cloud storage, in a different storage location than the Google login data.

Figure 5 shows the main windows and the basic workflow of the application.

### 2.2. Integrating Gyroscope and Accelerometer Sensors in the Application

**Figure 6:**
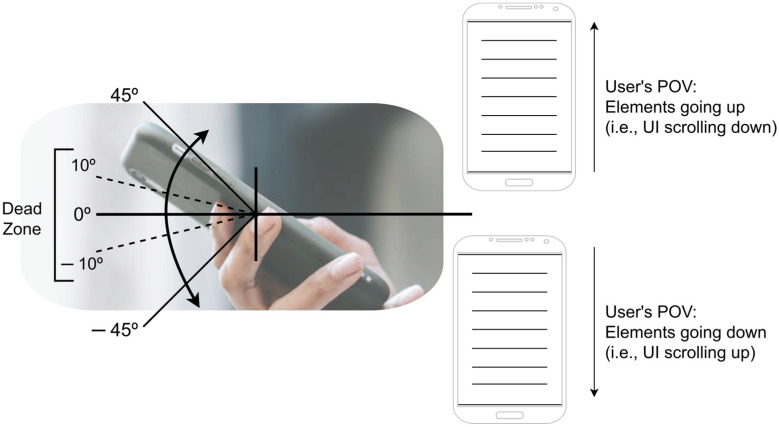
Concept image of sensor usage in “GyroList” module, gesture-based level. This level only features pitch motion.

The application developed employs a dynamic technique for using the accelerometer and gyroscope sensors to facilitate gesture-based interactions. For example, in the gesture-based level of the “GyroList” module, participants are prompted to tilt the mobile device forward (causing UI elements to move upward), and, respectively, tilting the device backward (causing a downward movement of the UI elements). The gyroscope sensor detects the tilting degree applied to the device, allowing the movement direction. Greater tilting degrees correspond to an increased speed in the on-screen movement. Simultaneously, the accelerometer sensor gauges the force of the tilt movement, thus influencing the impulse’s magnitude. That is, stronger force movements result in more pronounced on-screen speed, while slower movements yield lower GUI movement response. To enhance the user experience, a dead zone has been incorporated into the sensors’ detection mechanism. Within ten degrees from the baseline (both positive and negative pitch motion), no movement is registered, offering users a dead zone to facilitate a smoother and more controlled interaction with the application. This behavior is graphically explained in Figure 6.

As seen before, gesture-based levels involve dynamically detecting the tilting degree with the gyroscope sensor to determine the movement direction and speed. Simultaneously, the accelerometer contributes by assessing the force of the movement, modulating the magnitude of on-screen actions based on user’s input. There are modules with only vertical movement, and modules with both vertical and horizontal movements. Algorithm 1 shows the pseudocode of the algorithm for the vertical-only movement, applied in the “GyroList” module:
**Algorithm 1.** “GyroList” pseudocode for vertical-only movement.Accelerometer Data Processing: The value of the variable *x* is updated by subtracting the integer value of the X-axis accelerometer.Low-Pass Filter for Smoothing Y-coordinate (i.e., dead zone): The value of the variable *filteredY* is updated using a low-pass filter. The new value is a weighted sum of the current *filteredY* and the new accelerometer reading on the Y-axis. Variable *y* is also updated by subtracting the integer value of the filtered Y-coordinate. This is based on the following code:Low-pass filter: *filteredY = α·filtered +* (1 *− α*)·*event.values*[1] ^1^Update *y*: *y = y − filteredY.toInt*()Scrolling Behavior: The scrolling amount is determined based on the Y-coordinate of the filtered Y-coordinate, depending on whether the device is in a moving state (i.e., increasing or maintaining speed) or in a braking state (i.e., reducing speed). This scrolling is applied to the UI. Here is the used code:For moving: *scrollAmount =* (*−y*)*·sensitivity* ^2^For breaking: *scrollAmount =* (−*yDifference*)·*sensitivity* ^2^Filtering for Braking: For updating the filtered Y-coordinate for braking using a low-pass filter: Applied by the following code:Low-pass filter: *filtered =* (*α·filtered*) *+* ((1 *− α*)·*y*)Handling Previous Filtered Y-coordinate: *yDifference* is calculated as the difference between the current and previous filtered Y-coordinates. *previousFilteredY* is then updated with the current value. To achieve this, the following code is used:*yDifference = filtered − previousFilteredY*^1^ Array position for the desired reading from the sensor, as specified by Android SDK. ^2^ “*sensitivity*” is a constant value that we use to manually adjust the sensitivity of the scrolling behavior without changing the algorithm.

On the other hand, Algorithm 2 is the algorithm for the gesture-based levels with free movement, applied in the “Tutorial” “GyroAvoid” and “Survival” modules:
**Algorithm 2.** “Tutorial”, “GyroAvoid” and “Survival” pseudocode for free movement.Gyroscope and Accelerometer Data Processing: The position based on *x* and *y* variables is updated based on the data readings from the sensors by the following code:*newX = view.x* − (*event.values*[0] ^1^·*sensitivity* ^2^)*newY = view.y* + (*event.values*[1] ^1^·*sensitivity* ^2^)Updating View Position: UI’s *x* and *y* positions are updated with the previously calculated values:*view.x = newX.toFloat*()*view.y = newY.toFloat*()^1^ Array positions for the desired reading from the sensor, as specified by Android SDK. ^2^ “*sensitivity*” is a constant value that we use to manually adjust the sensitivity of the scrolling behavior without changing the algorithm.

## 3. Results

In this section, we present the outcomes of a pilot experimentation conducted with the Android application developed. The primary objective was to assess the feasibility and functionality of the app in capturing both quantitative (i.e., time) and qualitative (i.e., behavior) variables related to gesture-based and traditional touch-based interactions.

The pilot experimentation involved a group of 22 participants engaging with the app (see Table 1); recruited via random sampling within a college environment (no prerequisites were asked). We looked for valuable preliminary insights into the usability dynamics of gesture-based interactions. The results presented herein encapsulate the initial findings, demonstrating the practicality of our experimental design. They also open the way for a more comprehensive examination of the user experience in future studies.

In the pilot environment, participants were introduced to the primary objectives of the application, emphasizing the exploration of potential differences between using device sensors for gesture-based interactions and the traditional touch-based approach. With the aim of simulating real-world usage scenarios, participants were granted the freedom to navigate through the various levels and modules of the app independently. The minimal intervention from supervisors was intentional, as we sought to encourage participants to provide qualitative feedback based on their experiences, allowing us to improve the app for future studies. Prior to use the app, participants were informed about the anonymous treatment of generated data, ensuring that the information collected would not be associated with their personal identities. Verbal consent was obtained from each participant.

### 3.1. Analyzing Quantitative Data from the Pilot Study

**Figure 7:**
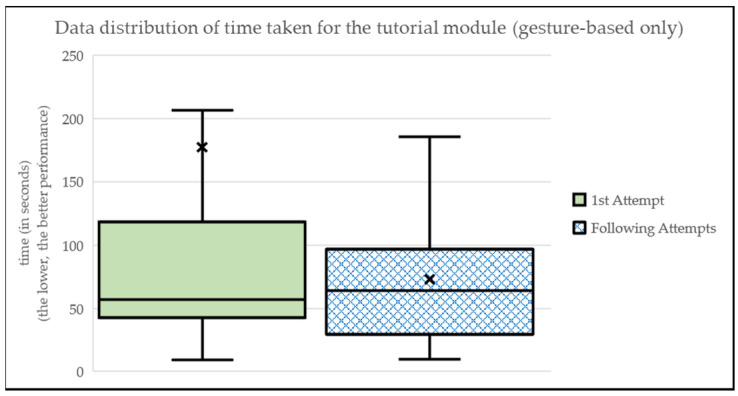
Data distribution of time taken for the tutorial module.

When analyzing the different times obtained in the “Tutorial” module (i.e., first activity of the app, a maze-like scenario with gesture-based level only) (see Figure 7 and Table 2), it can be seen that, in many cases, users have made a first attempt with a longer time (MEAN_FirstAttempt_ = 177.32; SD_FirstAttempt_ = 470.05) than in subsequent attempts (MEAN_FollowingAttempts_ = 73.04; SD_FollowingAttempts_ = 56.19). This may be due to the initial lack of knowledge about the use of sensors and the corresponding adaptation to them. Users were granted the freedom to explore and engage with the app at their own pace during the experimentation. Subsequent attempts mean any number of additional interactions beyond the first attempt, providing users with the flexibility to revisit and practice using the app. For the majority of users, we have captured between two and four subsequent attempts.

**Figure 8:**
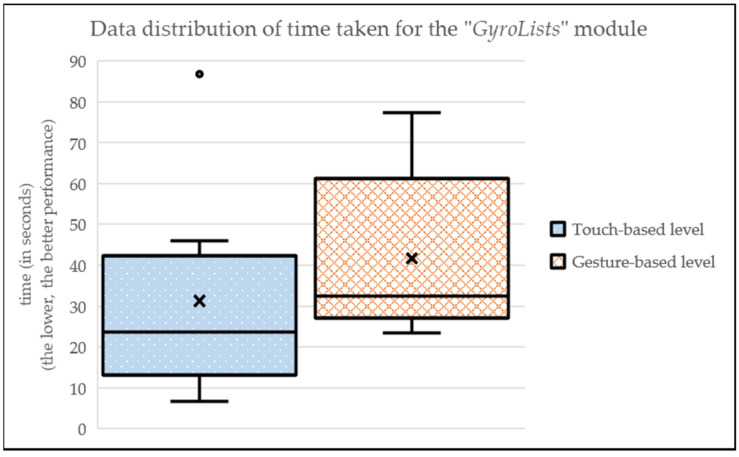
Data distribution of time taken for the “GyroLists” module.

Regarding the “GyrosLists” module (i.e., navigation through a list of elements), we can see that, in the touch-based level, the times obtained are slightly lower (MEAN_touch-based_ = 31.32; SD_touch-based_ = 25.49) than those obtained in the gesture-based level (MEAN_gesture-based_ = 41.73; SD_gesture-based_ = 20.33) (see Figure 8 and Table 2). That is, the traditional way is objectively more efficient for navigating through a list.

**Figure 9:**
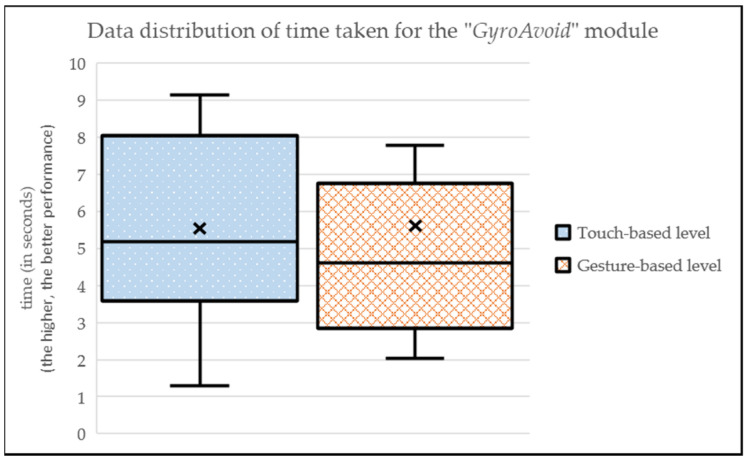
Data distribution of time taken for the “GyroAvoid” module.

As for corresponding to the “GyroAvoid” module (i.e., avoiding collisions with only left and right movements), we can see how both levels have quite similar timing records (Figure 9 and Table 2). We can conclude that both the touch-based (MEAN_touch-based_ = 5.54; SD_touch-based_ = 2.66) and the gesture-based (MEAN_gesture-based_ = 5.62; SD_gesture-based_ = 3.88) levels have the same performance in this game-like scenario.

**Figure 10:**
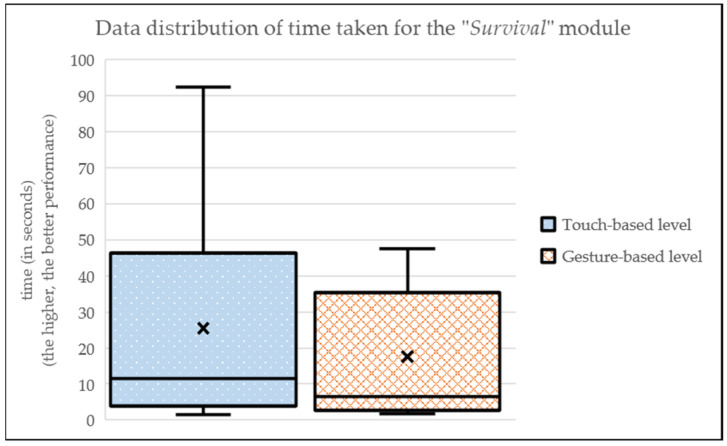
Data distribution of time taken for the “Survival” module.

Finally, the results for the “Survival” module (i.e., avoiding collisions with free movements), depicted in Figure 10 and Table 2, demonstrate a slightly better performance (in time) by users in the touch-based level (MEAN_touch-based_ = 25.44; SD_touch-based_ = 31.09). On the other hand, in the gesture-based level, there is a worse performance in the times for the participants (MEAN_gesture-based_ = 17.61; SD_gesture-based_ = 18.48).

### 3.2. Analyzing Qualitative Data from the Pilot Study

At the conclusion of each level, participants contribute to the quantitative analysis of user behavior through a post-task questionnaire. This questionnaire encompasses a twofold assessment, starting with a level rating on a Likert scale from 1 (very easy) to 5 (very hard), including intermediate points, gauging their perceived complexity of the level. Additionally, participants are encouraged to express their opinions freely through a commentary section. This open-text format allows participants to provide detailed insights, comments, and suggestions based on their individual experiences with the gesture-based and touch-based interactions.

**Figure 11:**
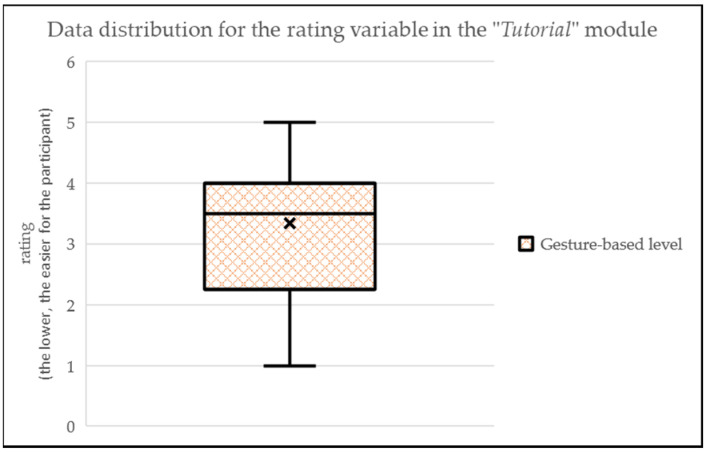
Data distribution for the rating variable in the “Tutorial” module.

The “Tutorial” module (i.e., first activity of the app, a maze-like scenario with gesture-based level only) shows that pilot participants value it in an intermediate complexity level (MEAN = 3.34; SD = 1.30) (see Figure 11). 23% of the participants noted that it is and interesting module to understand the possibilities of the device sensors. Also, 36% of participants highlighted that we need to smooth the precision in the movement when using the sensors, as sometimes it is difficult to move the ball precisely through the scenario if the device has to be placed at almost 90 degrees from the baseline position.

**Figure 12:**
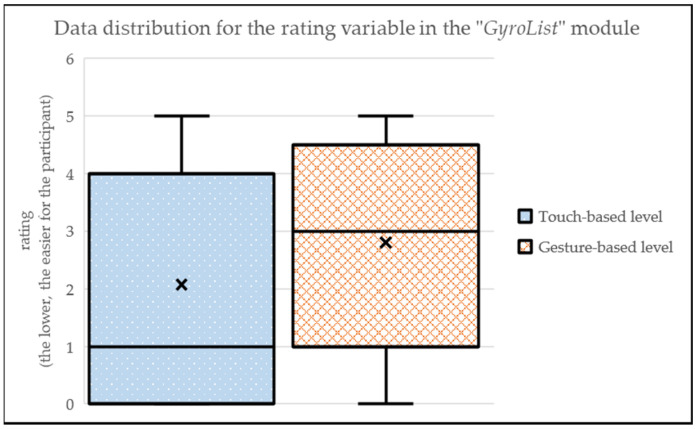
Data distribution for the rating variable in the “GyroList” module.

Regarding the “GyrosLists” module (i.e., navigation through a list of elements), the touch-based level has a lower perceived complexity (MEAN_touch-based_ = 2.07; SD_touch-based_ = 2.10) than the gesture-based level (MEAN_gesture-based_ = 2.80; SD_gesture-based_ = 1.78) to find the required element in the list (see Figure 12). This may be possibly due to a latent learning effect with the traditional touch-based ways of interacting with the GUI. As seen before, participants required less time to complete the touch-based level. This is reflected in the fact that 38% of the participants said that touch-based level was quick and easy. Nonetheless, the gesture-based level was said to be slower but a more convenient way to interact with a list of elements.

**Figure 13:**
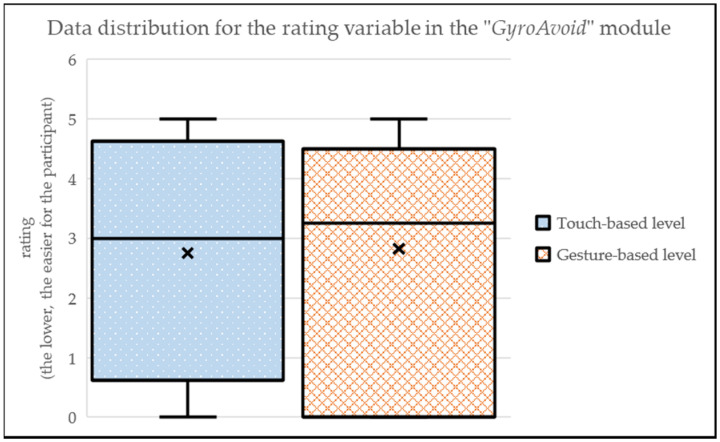
Data distribution for the rating variable in the “GyroAvoid” module.

The “GyroAvoid” module (i.e., avoiding collisions with only left and right movements), presents a rather similar perceived complexity, both in touch-based level (MEAN_touch-based_ = 2.75; SD_touch-based_ = 1.99) and in gesture-based level (MEAN_gesture-based_ = 2.82; SD_gesture-based_ = 1.96) (see Figure 13). The comments do not show a clear preference: 29% of participants liked more the gesture-based level, while 14% of participants preferred the touch-based level. In addition, like in other modules, comments encouraged to make the gesture-based interaction smoother for better interactions with the device sensors.

**Figure 14:**
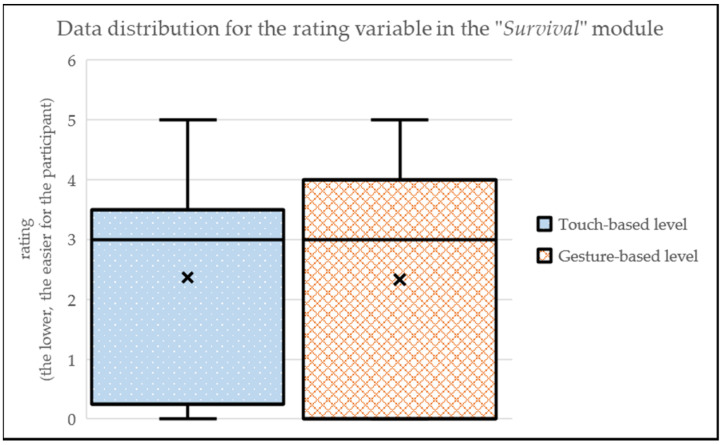
Data distribution for the rating variable in the “Survival” module.

Finally, in the “Survival” module (i.e., avoiding collisions with free movements), the touch-based level presents a similar perceived complexity (MEAN_touch-based_ = 2.36; SD_touch-based_ = 1.94) than the gesture-based level (MEAN_gesture-based_ = 2.33; SD_gesture-based_ = 1.98) (see Figure 14). The trend is similar to the previous module, maybe because both of these modules are game-like scenarios and the differences between forms of interactions is not so relevant in these cases. Similarly to the previous level, the comments do not show a clear preference: 18% of participants liked more the gesture-based level, while 27% of participants preferred the touch-based level.

## 4. Discussion

Gyroscope and accelerometer sensors are widely applied across diverse domains in mobile applications, and their effectiveness can vary significantly based on the specific tasks and applications. Consequently, it becomes challenging to draw generalizable conclusions or find direct equivalences among different types of research works.

De Sun et al. [12] created a support tool for gesture-based interaction design that connects gestures, actions, objects, and meanings through a database. They identified three key features for UX with gesture-based interactions: “Ensure basic functionality”, “enhance accessibility” and “assign meaning and value”. We believe our app is easy, intuitive and fulfills a need, thus covering all of these key features for this type of interaction. And, in some ways, looking at what people said, we can see that using sensors to navigate the UI is potentially attractive.

Chang et al. [33] applied the gyroscope and accelerometer sensors in an experiment to help reach screen targets with one hand. They lead to the design of three novel mobile interaction techniques, based on users’ natural tilting behavior with smartphones: “TiltSlide” (screen moved to a more accessible zone), “TiltReduction” (screen adjusted to a smaller size) and “TiltCursor” (use of a pointer controlled by the user’s finger). There are no similar implementations to ours in what these authors propose. While we move UI elements, the other authors move the screen. However, it should be noted that our application does not lose any of the visible or useful space of the screen. This is contrary to what these authors apply. Looking at the results, only TiltReduction (i.e., minimizing the usable screen area, thus no tilt is applied in the final interaction) showed better results than the traditional direct input. Somehow this coincides with what we found in our pilot results. However, following the authors ideas, we found our research valuable given that authors found that users tend to tilt the smartphone when the situation is uncomfortable (mainly, left side of the screen and the screen edges).

Gesture-based interaction activities presented in our app are not integrated within the typical mental activities with smartphones, so task complexity differs from traditional forms of interaction (i.e., touch based). Therefore, as derived from Banu, Al Siyabi and Al Minje [24], and Kosch et al. [25]; there is an expected higher cognitive load required on the users’ brain to complete new tasks. Perhaps this is why participants took longer to complete our gesture-based tasks. Authors pointed out that usability and standardization would help to a better relationship between users and gesture-based HCI.

Inherently, there is a novelty in our proposed gesture-based interaction usage. Touch-based interaction is the base form of interaction with smartphones. Our app (and, therefore, users) faces learnability effects, like first time performance or ability to remember skills over time [42]. In gesture-based interactions with sensors, users meet a first-time experience mixed with the known touch-based interaction skills. To perform under the same conditions as touch-based, there is a learning curve that gesture-based users must overcome. Therefore, the results that we have obtained are better (quantitative variable) in the touch-based scenarios. However, we can see the potential of this gesture-based interactions with smartphones, as we have seen in the opinions expressed by the participants (qualitative variable).

However, understanding gesture-based interactions still requires future work. The small sample size is the main limitation of the results shown here. This may impact the generalizability of the findings to a broader audience and real-world scenarios. Moreover, conclusions concerning long-term learning effects should be approached with caution, given that the experimentation conducted here did not exceed one hour per participant. Another limiting factor in discussing the significance of this study is the difficulty in comparing the results with the limited number of similar studies in the literature. While this study provides valuable insights into the integration of device sensors for enhanced use of mobile apps, it is true that scarcity of similar studies in the existing literature complicates the comparison and assessment of the results within the context of established knowledge.

## 5. Conclusions

In conclusion, this study presents a novel exploration of smartphone user experience (UX) through the integration of gyroscope and accelerometer sensors for gesture-based interactions with mobile User Interfaces (UIs). The development of an Android application allowed us to create a pilot experimentation, revealing intriguing insights into the usability dynamics of gesture-based interactions. Despite gesture-based tasks showing longer completion times than touch-based tasks, participants consistently perceived these gesture-based interactions as more appealing for UI interactions, suggesting a notable discrepancy between efficiency metrics and user perceptions. This dichotomy highlights the need for a deeper understanding of gesture-based UX beyond traditional touch-based interactions.

Moreover, the challenge of presenting a comprehensive discussion arises from the context-specific nature of gesture-based applications with these sensors, making it difficult to generalize findings across diverse research studies and the limited number of works. The limited similar literature underscores the novelty of our findings, as the absence of analogous research highlights our contribution to the field. While the small sample size and scarcity of comparable studies are limitations, this also emphasizes the pioneering nature of our exploration into gesture-based interactions, offering fresh perspectives and future research lines in the dynamic landscape of mobile app usage and usability.

This study contributes valuable considerations for the design and usage of mobile applications with sensors as a main interaction element. Moving forward, the field may benefit from tailored future work approaches that acknowledge the special feature of sensor applications in different domains.

First, conducting larger-scale studies with a more diverse participant pool could provide a more robust understanding of the generalizability of the observed trends and user preferences. This would involve engaging users demographically broader to ensure the applicability of the findings across various user profiles.

Second, investigate the long-term effects of gesture-based interactions by extending the study duration and assessing user experiences over an extended period. This approach would capture potential learning curves, usage patterns, and real-world scenarios.

In addition, it should be very important to extend and refine the developed Android application. Currently, the app has limited user cases tailored to browsing lists and avoiding failing blocks. It is required to add new modules to the app, each carefully designed to further investigate specific facets of gesture-based interactions—for example, back and forth navigation, interacting with contextual menus, or interacting with buttons, among other app interactions. Furthermore, a crucial expansion lies in the exploration of cross-platform integration, analyzing sensor usage in iOS and other platforms.

These potential avenues for future work aim to further refine our understanding of the complex interplay between sensors, user interactions, and mobile app design, contributing to the enhancement of user experiences in the evolving digital landscape.

## Figures and Tables

**Table 1:** Participant demographics.

Characteristic		Quantity (N = 22)	Percentage
Age	18–25	7	31.81%
	26–35	3	13.64%
	36–45	4	18.18%
	+46	8	36.36%
Gender	Masculine	11	50%
	Female	10	45.45%
	Other	1	4.55%
How old is the smartphone	Less than a year	5	22.73%
	1–2 years	3	13.64%
	3–5 years	11	50%
	More than 5 years	3	13.64%
Location	North America	0	0%
	South America	0	0%
	Europe	22	100%
	Asia	0	0%
	Africa	0	0%
	Oceania	0	0%
Have you used any app that uses sensors?	Yes	10	45.45%
	No	12	54.55%
Have you used any motion sensors?	Yes	9	40.91%
	No	13	59.09%
Have you use any VR headset?	Yes	11	50%
	No	11	50%
When was the last time you used these sensors?	Less than 6 months	4	18.18%
	6–12 months	1	4.55%
	1–2 years	1	4.55%
	+3 years	6	27.27%
	I have never used those sensors	10	45.45%

**Table 2:** Summary of statistics for all modules.

Module	Level	MEAN	SD	MEDIAN
Tutorial	1st Attempt	177.32 s	470.05 s	56.66 s
	Following Attempts (n = 14)	73.04 s	56.20 s	63.74 s
GyroList	Touch-based level	31.32 s	25.49 s	23.64 s
	Gesture-based level	41.73 s	20.34 s	32.40 s
GyroAvoid	Touch-based level	5.54 s	2.66 s	5.18 s
	Gesture-based level	5.62 s	3.88 s	4.62 s
Survival	Touch-based level	25.45 s	31.09 s	11.58 s
	Gesture-based level	17.61 s	18.48 s	6.43 s

## Data Availability

Data are contained within this article.
